# Aptamer-Conjugated Polydiacetylene Colorimetric Paper Chip for the Detection of *Bacillus thuringiensis* Spores

**DOI:** 10.3390/s20113124

**Published:** 2020-06-01

**Authors:** Chaoge Zhou, Taeyeong You, Huisoo Jang, Hyunil Ryu, Eun-Seon Lee, Mi-Hwa Oh, Yun Suk Huh, Sun Min Kim, Tae-Joon Jeon

**Affiliations:** 1 Department of Biological Engineering and Biohybrid Systems Research Center (BSRC), Inha University, 100 Inha-ro, Michuhol-gu, Incheon 22212, Korea; zhouchaoge1995@gmail.com (C.Z.); cmyu0816@gmail.com (T.Y.); huisoojang@gmail.com (H.J.); hyunil.ryu@gmail.com (H.R.); yunsuk.huh@inha.ac.kr (Y.S.H.); 2 National Institute of Animal Science, Rural Development Administration, Jeollabuk-do 55365, Korea; les1023@korea.kr (E.-S.L.); moh@korea.kr (M.-H.O.); 3 Department of Mechanical Engineering, Inha University, 100 Inha-ro, Michuhol-gu, Incheon 22212, Korea

**Keywords:** polydiacetylene (PDA), *Bacillus thuringiensis* spores, aptamer, paper chip, chromatic sensor

## Abstract

A colorimetric polydiacetylene (PDA) paper strip sensor that can specifically recognize *Bacillus thuringiensis* (BT) HD-73 spores is described in this work. The target-specific aptamer was combined with PDA, and the aptamer-conjugated PDA vesicles were then coated on polyvinylidene fluoride (PVDF) paper strips by a simple solvent evaporation method. The PDA-aptamer paper strips can be used to detect the target without any pre-treatment. Using the paper strip, the presence of BT spores is directly observable by the naked eye based on the unique blue-to-red color transition of the PDA. Quantitative studies using the paper strip were also carried out by analyzing the color transitions of the PDA. The specificity of this PDA sensor was verified with a high concentration of *Escherichia coli*, and no discernable change was observed. The observable color change in the paper strip occurs in less than 1 h, and the limit of detection is 3 × 10^7^ CFU/mL, much below the level harmful to humans. The PDA-based paper sensor, developed in this work, does not require a separate power or detection device, making the sensor strip highly transportable and suitable for spore analysis anytime and anywhere. Moreover, this paper sensor platform is easily fabricated, can be adapted to other targets, is highly portable, and is highly specific for the detection of BT spores.

## 1. Introduction

Certain bacteria can form durable, self-protective spore structures that can survive under harsh conditions. Eliminating bacterial spores with conventional sterilization methods is very difficult because the spore structure can protect it from environmental stresses, including desiccation, high temperature, UV irradiation, enzymatic destruction by drugs and exposure to toxic chemicals [1]. Therefore, the simple and rapid detection of spores is a widespread concern in food safety. In previous studies, polymerase chain reaction (PCR) [2,3], immunoassays [4], fluorescence assays using quantum dots [5], and counting methods for *Bacillus* spores have been demonstrated. However, sophisticated methods and non-transportable instruments have limited their use in the field. Therefore, we developed a simple and transportable paper sensor platform to detect well-known spores from the phylum Firmicutes, *Bacillus thuringiensis* (BT) spores. BT spores form crystal proteins that are toxic to numerous insects and other invertebrates [6,7]. BT spores are generally recognized as biosafe insecticides and are toxic to a human at extreme doses (approximately > 10^11^ CFU/mL) [5,6,8]. However, the biosafety of BT spores has been controversial, as previous reported, and they have some physiological effects that may be infectious [9]. Thus, BT spores can be good examples for testing our versatile sensing platform, while they are relatively safe to handle in the typical laboratory settings.

In this work, we conjugated a target-specific aptamer with polydiacetylene (PDA) sensor for the chromatic detection of spores that avoids the limitations of previous methods. Polydiacetylene (PDA) is an excellent material, often used in colorimetric biosensors, due to its unique chromatic properties, which cause color changes that are visible to the naked eye. A monomer, diacetylene (DA), can be readily polymerized into a blue polydiacetylene polymer under ultraviolet light irradiation (λ = 254 nm). Cross-linked PDAs show a blue–red colorimetric transition, when external stress stimulates the backbone through the pendant side chains, in response to pH, temperature, mechanical or chemical stresses [10]. Furthermore, these chromatic changes can be triggered by the binding of a target analyte with sensing probes such as peptides [11,12], DNA aptamers [13,14], or antibodies [15] conjugated to the PDA pendant side chains. In particular, DNA aptamers are produced by chemical synthesis and offer high stability and affinity [16]. The properties of aptamers can be easily designed on demand [16].

In general, analyte-specific PDA sensors have been studied in the form of liposomes [17,18], Langmuir–Blodgett films [19], silica beads [20], and strips [21,22,23]. Among those methods, paper strips provide a suitable platform for simple, real-time testing [24,25]. A paper strip combined with the color transition characteristics of PDA provides directly observable results based on its color change. Seo et al. developed a paper strip sensor that can detect spores based on Eu^III^-EDTA conjugated PDA [26]. However, they did not detect a specific spore but only the binding of calcium dipicolinate (DPA), which is a major component of various bacterial endospores. For this reason, this sensor system has no specificity between different spores.

**Figure 1:**
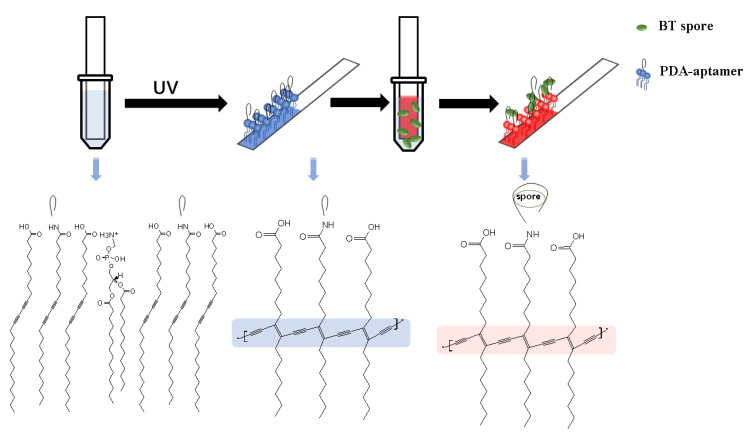
Schematic of the PDA-aptamer paper strip coating, the entire detection process and the key chemical structures. The immobilized PDA-aptamer was polymerized by UV exposure, and it turned blue. After a short incubation period, the BT spores were recognized by the PDA-aptamer, and the reaction between the aptamer and spore induced a color change in the PDA from blue to red.

To specifically detect a target spore, *Bacillus thuringiensis* spore, we conjugated a target-specific aptamer with polydiacetylene through an EDC-NHS reaction. The aptamer-conjugated PDA was subsequently coated on a polyvinylidene fluoride (PVDF) paper strip by a simple solvent evaporation method. The immobilization of PDA on the paper strip enhanced its color response by a factor of over 100 compared to that of PDA vesicles suspended in solution, which is consistent with other reports and has been exploited for microorganism detection [27]. After immersing the paper sensor in the samples with no additional sample preparation steps, due to the bichromic characteristics of polydiacetylene, the aptamer-modified PDA paper sensor showed a remarkable color transition upon exposure to BT spores in solution (Figure 1). The visible color change of the paper strip occurred in less than 1 h, and the detection limit of BT spores is as low as 3 × 10^7^ CFU/mL. Moreover, depending on the concentration of the spore sample, quantitative analysis could be achieved based on the degree of the color change. The PDA-based paper sensor developed in this work does not require a separate power supply or detection device, making the paper-based sensor strip transportable for the facile analysis of spores anytime and anywhere. In addition, based on the versatility of aptamers, other deleterious Bacillus species, such as *B. anthracis* and *B. cereus* that cause diseases including foodborne illnesses and tissue necrosis [28,29], can be identified by using different probe aptamers on the paper strips.

## 2. Materials and Methods

### 2.1. Materials

10,12-Tricosadiynoic acid (TCDA) (91445), 10,12-pentacosadiynoic acid (PCDA) (76492), N-(3-dimethylaminopropyl)-N’-ethylcarbodiimide hydrochloride (EDC-HCl) (E6383), ethanolamine (411000), N-hydroxysuccinimide (NHS) (130672), phosphate-buffered saline powder (P3813), methylene chloride and chloroform were purchased from Sigma–Aldrich (St. Louis, MO, USA) and 99% HEPES (A14777) was purchased from Alfa Aesar (Ward Hill, MA, USA) (http://www.alfa.com). Dimethyl-2-(dimethylphosphino)ethylphosphine (DMPE) was purchased from Avanti Polar Lipids (Alabaster, AL) (http://www.avantilipids.com). BT spore specific aptamers were prepared, as described previously [5]. A sixty base 5′-amine modified oligonucleotide (5′-NH_2_-(CH_2_)_6_ -CAT CCG TCA CAC CTG CTC TGG CCA CTA ACA TGG GGA CCA GGT GGT GTT GGC TCC CGT ATC-3′) were obtained from Integrated DNA Technologies (IDT). The water used in all experiments was doubly distilled.

### 2.2. Preparation of the Bacillus Thuringiensis Spores

*Bacillus thuringiensis* spores were cultured following a previously described report [30]. Briefly, *B. thuringiensis* (BT, ATCC 35866) was incubated in nutrient broth solution and cultured for 24 h in Glucose Yeast Salt medium (GYS medium, Nickerson et al., 1974) [31]. After centrifugation and washing, the spore stock solution was prepared in PBS (pH 7.5, 5 mM). The formation of spores was observed with a microscope (TE2000-U, Nikon, Tokyo, Japan) [Figure S1]. The CFU value of the stock solution was determined by spreading diluted stock solutions on nutrient agar plates and incubating at 37 °C for 24 h and counting the colonies formed. The final concentration of the culture was determined to be 3 × 10^11^ CFU/mL by colony counting, and the prepared spore solution was stored at −20 °C until use to prevent additional growth.

### 2.3. Preparation of TCDA-NHS

Conversion of TCDA to succinimidyl active esters was carried out as previously reported [22]. Briefly, 0.25 g (0.72 mmol) of TCDA, 0.26 g (1.35 mmol) of EDC-HCl and 0.12 g (1.07 mmol) of NHS were dissolved in 4 mL of methylene chloride. After stirring the solution gently with a magnetic stirrer for 2 h at room temperature, the solvent was evaporated with a stream of argon. To purify the evaporated residue, the material was extracted with ethyl acetate using a separation funnel, TCDA-NHS monomer was obtained as a white solid. ^1^H nuclear magnetic resonance (NMR) (400 MHz, chloroform-d) δ ppm 0.86 (t, 3 H), 1.24–1.79 (m, 28 H), 2.22 (t, 4 H), 2.58 (t, 2 H), 2.81 (s, 4 H) (Figure S2).

### 2.4. Preparation of PDA Liposomes for Bacillus Thuringiensis Spore Detection

To conjugate the amine aptamer with TCDA-NHS, 900 nmol TCDA-NHS (0.41 mg) was mixed with the aptamer (16 µL) in 20 µL DMSO and reacted for 4 h at 37 °C. The BT spore binding aptamer used here was developed by M. Ikanovic et al. using a SELEX (Systematic Evolution of Ligands by EXponential Enrichment) method. In this work, the selectivity of the aptamer was further tested with another BT strain, *Bacillus globigii* as a negative control. There was no noticeable increase of fluorescence intensity when the aptamer was treated with *Bacillus globigii* at the concentration of 10^6^ CFU/mL [5]. The unreacted succinimidyl active esters were inactivated by the addition of ethanolamine. The unreacted succinimidyl active esters were inactivated by the addition of ethanolamine. PCDA/DMPE was mixed at a ratio of 1:1 in chloroform, and the solvent was evaporated by a stream of argon gas. The PCDA and DMPE mixture was re-suspended in HEPES buffer (5 mM, pH = 7.5) to a total lipid concentration of 3 mM in a glass vial and heated for 1 h in a water bath (WiseBath, WB-6) at 80 °C. A preheated solution of TCDA-aptamer in DMSO was added, and the PDA-aptamer solution was sonicated for 10 min (Sonics VCX-750 Vibra Cell Ultra Sonic Processor). The sonicated solution was filtered through a 0.8-μm syringe filter (Advantec dismic-25cs) and stored at 4 °C overnight. The liposome solution was irradiated with 254 nm UV light for 5 min to yield a blue color.

### 2.5. Preparation of a PDA Paper Sensor for Bacillus Thuringiensis Spore Detection

To fabricate the BT spore-specific PDA sensor strip, the aptamer was conjugated with TCDA-NHS by the method described earlier. PCDA was mixed with DMPE at a ratio of 1:1 in chloroform, and then the TCDA-aptamer was dissolved in 800 µL of chloroform to a total lipid concentration of 3 mM, including 5% aptamer. The PVDF paper was prepared in 1 cm × 5 cm rectangles. To uniformly coat the PDA-aptamer on the surface of the paper strip, the prepared paper was quickly dipped in the PDA-chloroform solution and immediately removed. After drying at room temperature, the PDA on paper strip was polymerized under 254 nm UV radiation for 45 s to yield a blue color (Figure 1).

### 2.6. Incubating the PDA Paper Strips in Bacillus Thuringiensis Spore Solution

Stock BT spore solutions were prepared at concentrations of 3 × 10^7^, 3 × 10^8^, 3 × 10^9^, 3 × 10^10^ and 3 × 10^11^ CFU/mL, and then the PDA-aptamer-coated paper strips were dipped into the BT spore solutions for 4 h. Images of the paper strips were recorded every hour.

## 3. Results and Discussion

### 3.1. Detection of BT Spores with PDA-Aptamers Suspended in Solution

BT spores can kill insects and cause tremendous damage to silk farms. However, these bacteria are nontoxic to humans, and thus, they can be studied without infection risk. To detect spores, a BT-specific aptamer was used as a bio-receptor, due to its stability and tunability. Moreover, aptamer-based sensing systems can be expanded to other targets by using target-specific aptamers. In this experiment, an aptamer with a hairpin-shaped structure was used to increase the detection capacity [32]. Moreover, to further demonstrate the concept of a colorimetric aptamer-based paper strip sensor, a BT spore was chosen as the target since it can be easily obtained and relatively safe to handle in the laboratory.

First, we conjugated the BT-specific aptamers on PDA. The conjugated PDA vesicles were prepared using a conventional method [33]. To verify the most efficient ratio between PDA and the DMPE lipid mixture, we first prepared PDA-lipid mixture liposomes for detecting BT spores. Finally, we decided to use a TCDA-aptamer/PCDA/DMPE mixture with a PCDA to DMPE molar ratio of 1:1 [13]. DMPE was added to the diacetylene monomers to increase the sensitivity of the PDA liposomes.

After preparation, the aptamer-conjugated PDA vesicles were incubated BT spore solutions with different concentrations for 4 h. The target spores captured by the PDA vesicles produced steric repulsion at the PDA liposome surface, resulting in perturbations of the PDA ene−yne backbone. As a result, the PDA vesicles changed color. The color transition of the vesicles was monitored with a microplate absorption spectrophotometer, and the color response (CR), which characterizes the percent conversion of the liposomes from blue to red, was calculated as in previous literature [34]. The percent blue (PB) is defined by the following equation,

$$PB=\frac{A_{blue}}{A_{blue}+A_{red}}\times 100\%$$

where $A_{blue}$ is the absorption at 620 nm (blue form of the liposome), and $A_{red}$ is the absorption at 520 nm (red form of the liposome). Then, CR is defined as,

$$CR=\frac{PB_{0}-PB_{f}}{PB_{0}}\times 100\%$$

where $PB_{0}$ is the percent blue of the PDA liposomes before adding the BT spores and $PB_{f}$ is the final percent blue after detection.

**Figure 2:**
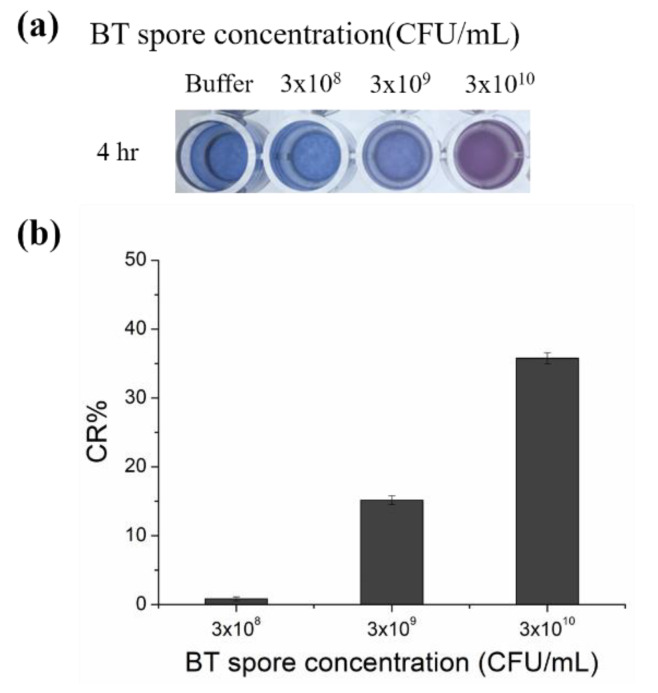
PDA-aptamer liposomes incubated with different concentrations of spore solutions. (**a**) Image of PDA-aptamer liposomes after 4 h of incubation in PBS (pH = 7.5, 5 mM) and spore solutions at different concentrations. (**b**) Color response percentage (CR%) of the PDA-aptamer liposomes after 4 h of incubation in spore solutions of different concentrations. Color response data are presented as the mean ± SD (n = 3).

Figure 2b shows the colorimetric responses to the target spores with PDA vesicles suspended in solution. No obvious color change (%CR) occurred below a concentration of 3 × 10^8^ CFU/mL spores in 4 h of incubation.

### 3.2. Detection of BT Spores with PDA-Aptamer Immobilized on PVDF Paper Strips

**Figure 3:**
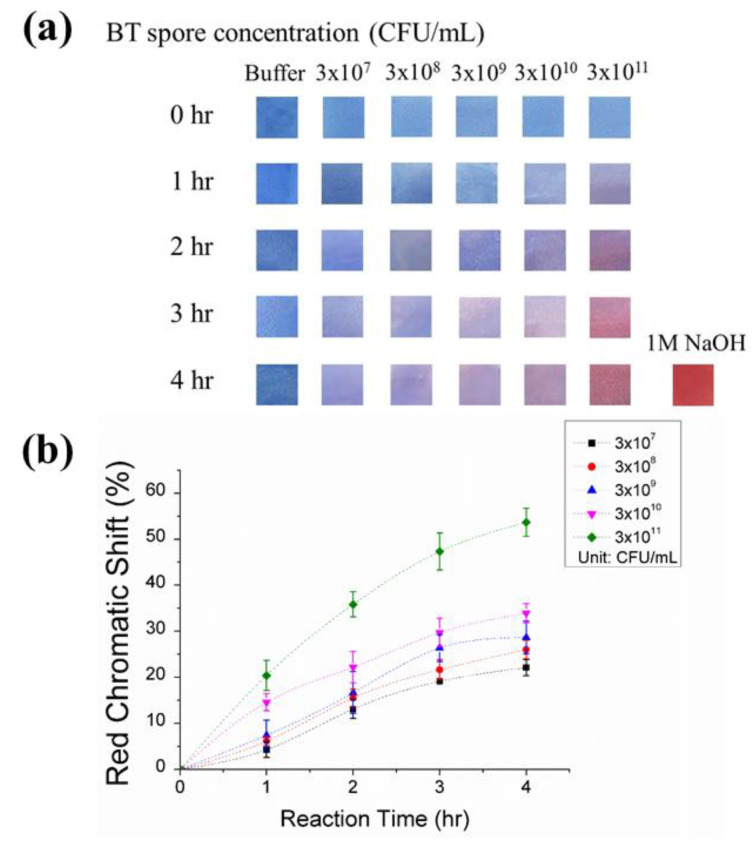
PDA strip detection of BT spores. (**a**) Images of the paper strips after incubation in spore solutions of different concentrations for 4 h. The negative and positive control images are from strips dipped in PBS (5 mM, pH = 7.5), and 1 M NaOH, respectively. (**b**) Red chromatic shift of the strips after 1 h, 2 h, 3 h, and 4 h of incubation in the spore solutions. Red chromatic shift data are presented as the mean ± SD (n = 3).

The PDA-aptamer-coated paper strips were incubated in BT spore solutions at concentrations of 3 × 10^7^, 3 × 10^8^, 3 × 10^9^, 3 × 10^10^, and 3 × 10^11^ CFU/mL for 4 h, and images of paper strips were taken every hour. A slight color transition could be observed by the naked eye after 1 h, and more dramatic color changes were observed as the incubation time was increased (Figure 3). To quantify the red chromatic shift percent, the images of the paper strips were analyzed by ImageJ software to obtain image-averaged red-green-blue (RGB) values [Table S1]. The mean value of the RGB data from ImageJ was used to calculate the red chromaticity level (r) as previously described [32]:

$$r=\frac{R}{R+G+B}.$$

The percent extent of the blue-to-red transition is defined as red chromatic shift (RCS) and was determined by

$$RCS=\frac{r_{sample}-r_{0}}{r_{max}-r_{0}}\times 100\%.$$

Here, $r_{sample}$ is the average red chromaticity level of spore solution-incubated paper strips, $r_{0}$ is the average red level of buffer-incubated paper strip (negative control), and $r_{max}$ is the average red chromaticity level of the maximal blue–red transition, usually the blue–red color transition of the paper in 1 M NaOH (positive control).

The RCS analysis indicated that the extent of the blue-to-red transition increased with exposure time. With samples at 3 × 10^11^ CFU/mL, the RCS increased to 20.4% in the first 1 h and to 35.8% after 2 h. After 4 h of incubation, the RCS reached 53.7%, the highest value in all the tested samples. This PDA-based aptamer probe on a paper strip allows the semi-quantitative analysis of BT spores after 4 h of incubation. At different concentrations of spores, the paper strips demonstrated quantitative RCS% values of 22.1%, 26.0%, 28.6%, 33.9%, and 53.7% RCS for 3 × 10^7^, 3 × 10^8^, 3 × 10^9^, 3 × 10^10^, and 3 × 10^11^ CFU/mL, respectively. Moreover, the color transitions in all the samples were observable with the naked eye, enabling on-site detection of the presence of spores.

Our paper strip sensor showed a LOD of 3 × 10^7^ CFU/mL with a 4 h reaction time. Compared to the LOD of the free suspended vesicle solution (Figure 2), the immobilization of the PDA sensor on the paper strip resulted in better sensitivity (increased by a factor of 100) for the following reasons. First, the solid-phase fixed PDA provided more rigidity so that the stresses of the target spores more effectively disturbed and triggered the backbone. Second, the membrane in solution can concentrate analytes and prevent analyte diffusion [27]. The BT spores targeted in this work are reported to be harmful to humans at high doses (approximately >10^11^ CFU/mL). Therefore, our PDA paper sensor has sufficiently high sensitivity to determine risks, due to BT spores present in liquid samples. In our previous research, a protein nano-pore system that used the same sequence of BT spore aptamer showed the lower detection limit (1.2 × 10^1^ CFU/mL) than our paper strip [30]. However, no significant results were obtained at 1.2 × 10^7^ CFU/mL and higher concentration due to the depletion of unbound hairpins, precluding the use of the aptamer-based sensing system at relatively high concentration. Moreover, the sensor system, developed in the previous work, was designed for use in extremely low concentration of samples where a highly sensitive detection system is required, thus expertise is needed and onsite use is also limited. However, our paper strip provides versatile, simple, and portable platform that is compatible with field testing. To the best of our knowledge, PDA strips are stored in the dark and are able to detect the target after the 28 day period unless it is exposed by light [32]. PDA vesicles and aptamers usually do not change their properties for months in laboratory settings. Moreover, the solid-phase supported sensor will enable portability, long-term storage, and operational simplicity specifically for point-of-care detection.

### 3.3. Specificity of the PDA-Aptamer Paper Strip

**Figure 4:**
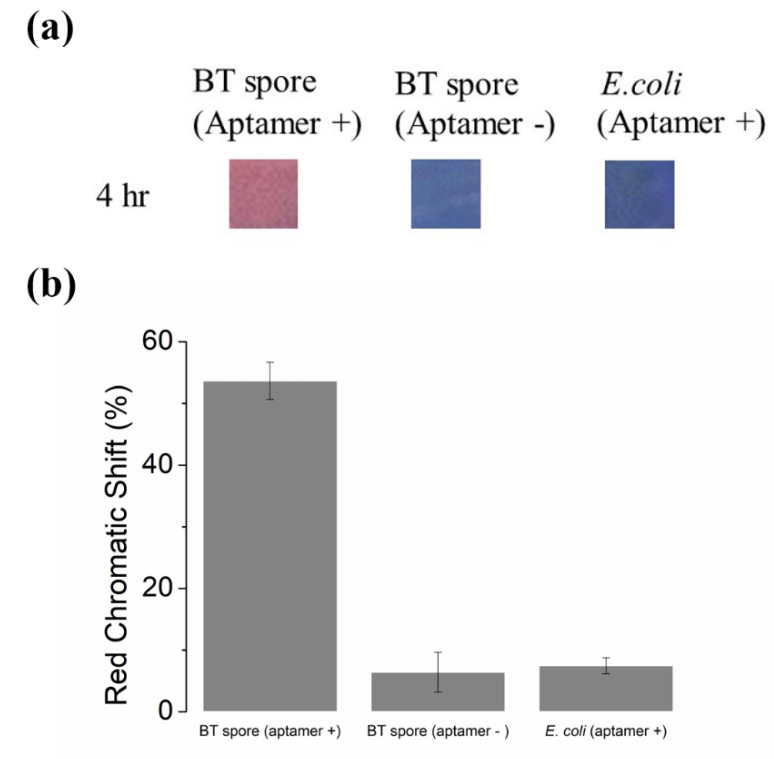
Testing the specificity of the PDA strips. (**a**) Images of the paper strips (with aptamer) after incubation in 3 × 10^11^ CFU/mL BT spore solutions, paper strip (without aptamer) in 3 × 10^11^ CFU/mL BT spore solution, and paper strip (with aptamer) in 3 × 10^11^ CFU/mL E. coli for 4 h. The negative and positive control images are strips dipped in PBS (5 mM, pH = 7.5) and 1 M NaOH. (**b**) Red chromatic shift of the strips after 4 h of incubation. Red chromatic shift data are presented as the mean ± SD (n = 3).

To verify that the signal from the PDA paper strip is specifically in response to BT spores, we prepared two experimental control groups for comparison. We incubated a PDA-aptamer conjugated paper strip in 3 × 10^11^ CFU/mL E. coli solution and a PDA-only paper strip (without target-specific aptamers) with 3 × 10^11^ CFU/mL BT spore solution. All experiments were performed multiple times, and pictures of the control strips were taken after 4 h of incubation and analyzed based on the method described above. In both the positive and negative control experiments, no significant color transitions occurred, showing that our paper strip specifically targets BT spores (Figure 4). Moreover, our sample was directly taken from the BT spore culture media with no pre-treatment or purification process, and it already contains a large amount of impurities as in real world samples, including the byproducts of GYS medium. Therefore, we assumed that our sensing platform would work with the real-world samples containing a large amount of impurities. In addition, by changing the aptamer to those targeting other spores or pathogens, our sensor strategy provides a versatile platform for detecting other bacteria, such as B. anthracis and B. cereus that are harmful to humans.

## 4. Conclusions

In this study, we devised an aptamer-based paper strip sensor for the detection of BT spores, based on the chromatic changes of the strip, enabling inexpensive preparation and good portability. The visible color transition of the PDA obviates the need for sophisticated protocols and instruments and provides simple sensors suitable for on-site testing. Moreover, there is no cumbersome or complicated pre-processing in our system, and the prepared PDA-aptamer paper strip can be used for immediate detection, as long as the targets are present in a liquid. The results of our system showed that PDA-aptamer paper-based strips exhibit varying degrees of color changes, depending on the concentration of BT spores and the detection time. Therefore, our sensor can be used for quantitative analysis, and the color changes are observable by the naked eye. In addition to rough visual observation, photos of the paper strips can also be evaluated through mobile programs to obtain more detailed and accurate quantitative data. The experimental results also showed the specificity of the sensor, and it has a low possibility of being affected by other foreign substances; thus, we can ensure that most of the color transition is caused by the target BT spores. By simply replacing the probe, or the aptamer, this sensor system is quite versatile and can be used to detect other pathogens such as viruses, bacteria and fungi. Although the detection limit of this system can be an issue, the sensor was able to detect spores on the spot using a piece of paper. In the future, along with improving the detection limit, we believe that this paper strip sensor can be widely used in on-site testing and will play an important role in food safety, water source safety and transportation safety where needed.

## Supplementary Materials

The following are available online at https://www.mdpi.com/1424-8220/20/11/3124/s1, Figure S1: Microscopic images of *Bacillus thuringiensis* (a) and GYS medium cultured *Bacillus thuringiensis* spores (b). The formation of spores can be confirmed by shape difference, Figure S2: ^1^H nuclear magnetic resonance (NMR) spectra of TCDA-NHS (400 MHz, chloroform-d) ∂ ppm 0.86 (t, 3 H) 1.24 - 1.79 (m, 28 H) 2.22 (t, 4 H) 2.58 (t, 2 H) 2.81 (s, 4 H); 2.81 (s, 4 H) indicates ^1^H peak of N-hydroxysuccinimide (NHS), Table S1: The RGB values of paper strips after 4hr incubation in different *Bacillus thuringiensis* spore concentrations.
