# Supplementary material for: Aptamer-Conjugated Polydiacetylene Colorimetric Paper Chip for the Detection of Bacillus thuringiensis Spores
Source: Sensors (Basel). 2020 Jun 1;20(11):3124. doi: 10.3390/s20113124 (PMC7308844; doi:10.3390/s20113124)
Supplement: Supplementary file 1 [file sensors-20-03124-s001.pdf]

sensors

Article

Aptamer-Conjugated Polydiacetylene Colorimetric Paper Chip for the Detection of *Bacillus thuringiensis* Spores

Chaoge Zhou [1], Taeyeong You [1], Huisoo Jang [1], Hyunil Ryu [1], Eun-Seon Lee [2], Mi-Hwa Oh [2], Yun Suk Huh [1], Sun Min Kim [3,*] and Tae-Joon Jeon [1,*]

[1] Department of Biological Engineering and Biohybrid Systems Research Center (BSRC), Inha University, 100 Inha-ro, Michuhol-gu, Incheon 22212, Korea; zhouchaoge1995@gmail.com (C.Z.); cmyu0816@gmail.com (T.Y.); huisoojang@gmail.com (H.J.); hyunil.ryu@gmail.com (H.R.); yunsuk.huh@inha.ac.kr (Y.S.H.)

[2] Animal Production Research and Development Division, National Institute of Animal Science, Jeollabuk-do 55365, Korea; les1023@korea.kr (E.-S.L.); moh@korea.kr (M.-H.O.)

[3] Department of Mechanical Engineering, Inha University, 100 Inha-ro, Michuhol-gu, Incheon 22212, Korea

* Correspondence: sunmk@inha.ac.kr (S.M.K.); tjjeon@inha.ac.kr (T.-J.J.); Tel.: +82-32-860-7328 (S.M.K.); +82-32-860-7511 (T.-J.J.); Fax: +82-32-877-7328 (S.M.K.); +82-32-872-4066 (T.-J.J.)

Received: 14 April 2020; Accepted: 29 May 2020; Published: 1 June 2020

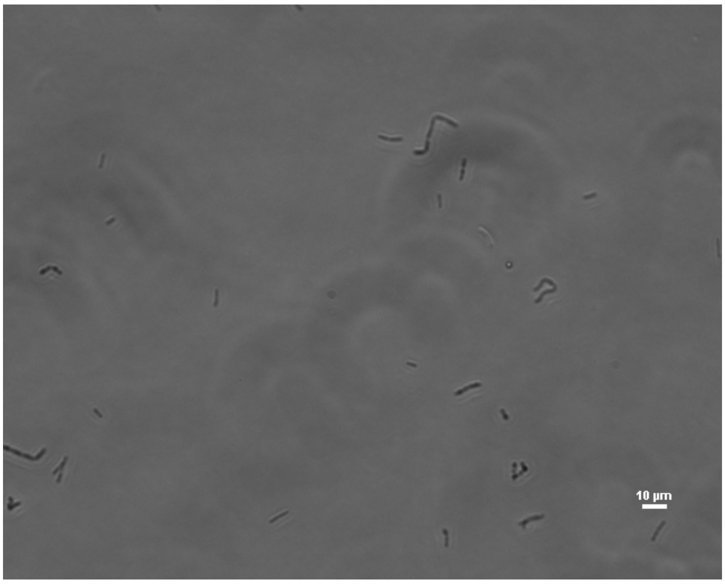

(a)

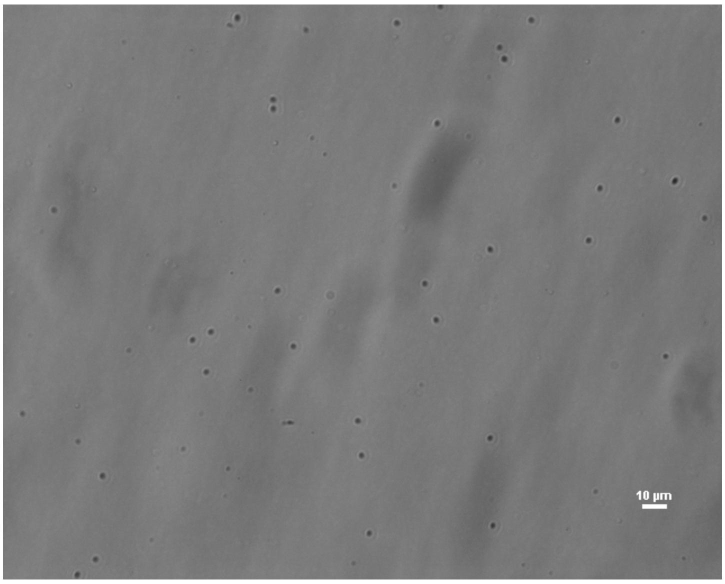

(**b**)

Figure S1. Microscopic images of *Bacillus thuringiensis* **(a)** and GYS medium cultured *Bacillus thuringiensis* spores; **(b)** The formation of spores can be confirmed by shape difference.

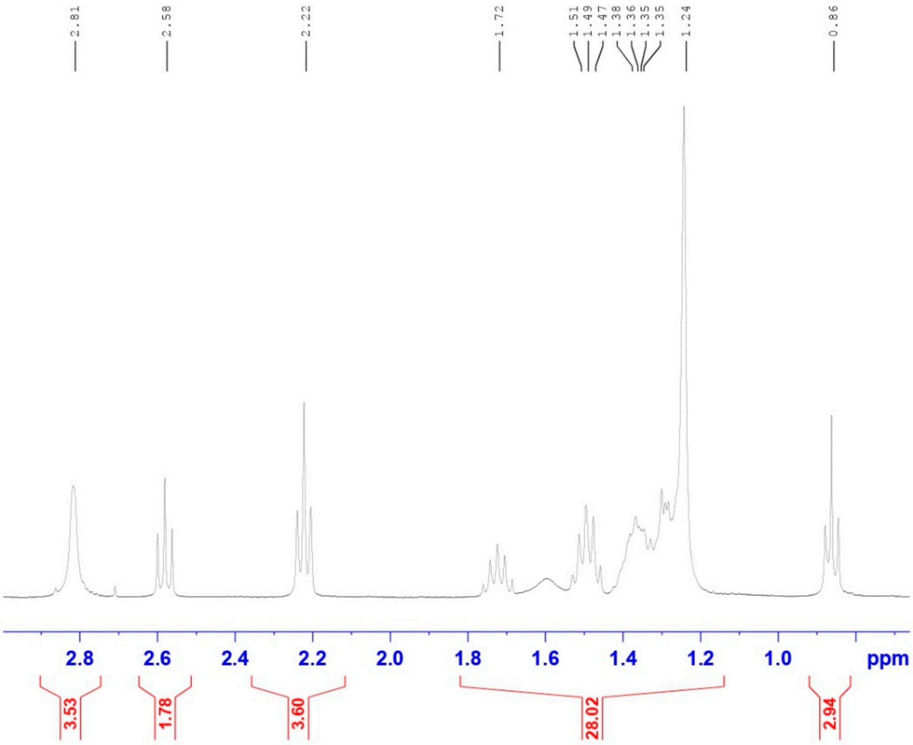

Figure S2. ^{1}H nuclear magnetic resonance (NMR) spectra of TCDA-NHS. (400 MHz, chloroform-d) ∂ ppm 0.86 (t, 3 H) 1.24–1.79 (m, 28 H) 2.22 (t, 4 H) 2.58 (t, 2 H) 2.81 (s, 4 H); 2.81 (s, 4 H) indicates ^{1}H peak of N-hydroxysuccinimide (NHS).

Table S1. The RGB values of paper strips after 4 h incubation in different *Bacillus thuringiensis* spore concentrations.

Sample (CFU/ml)	Red	Green	Blue
$3 \times 10^{7} - 1$	154.435	154.490	208.898
$3 \times 10^{7} - 2$	82.666	116.423	162.158
$3 \times 10^{7} - 3$	205.192	133.755	119.921
$3 \times 10^{8} - 1$	177.115	162.176	202.169
$3 \times 10^{8} - 2$	87.293	100.973	162.158
$3 \times 10^{8} - 3$	137.705	121.271	138.726
$3 \times 10^{9} - 1$	179.809	157.424	191.787
$3 \times 10^{9} - 2$	101.524	105.638	140.92
$3 \times 10^{9} - 3$	205.192	133.755	119.921
$3 \times 10^{10} - 1$	175.550	145.488	173.686
$3 \times 10^{10} - 2$	135.205	107.468	143.350
$3 \times 10^{10} - 3$	164.303	151.722	169.155
$3 \times 10^{11} - 1$	178.749	111.172	129.571
$3 \times 10^{11} - 2$	101.524	105.638	140.920
$3 \times 10^{11} - 3$	151.215	121.017	129.273

© 2020 by the authors. Licensee MDPI, Basel, Switzerland. This article is an open access article distributed under the terms and conditions of the Creative Commons Attribution (CC BY) license (http://creativecommons.org/licenses/by/4.0/).
